# Application of quality-by-design for adopting an environmentally green fluorogenic determination of benoxinate hydrochloride in eye drops and artificial aqueous humour

**DOI:** 10.1038/s41598-023-35347-6

**Published:** 2023-05-26

**Authors:** Mohamed A. El Hamd, Mahmoud El-Maghrabey, Galal Magdy, Wael A. Mahdi, Sultan Alshehri, Amr K. A. Bass, Hany A. Batakoushy

**Affiliations:** 1 Department of Pharmaceutical Sciences, College of Pharmacy, Shaqra University, Shaqra, 11961 Saudi Arabia; 2 Department of Pharmaceutical Analytical Chemistry, Faculty of Pharmacy, South Valley University, Qena, 83523 Egypt; 3 Department of Pharmaceutical Analytical Chemistry, Faculty of Pharmacy, Mansoura University, Mansoura, 35516 Egypt; 4 Pharmaceutical Analytical Chemistry Department, Faculty of Pharmacy, Kafrelsheikh University, Kafrelsheikh, 33511, Egypt; 5 Department of Pharmaceutics, College of Pharmacy, King Saud University, Riyadh, 11451 Saudi Arabia; 6 Department of Pharmaceutical Chemistry, Faculty of Pharmacy, Menoufia University, Shebin Elkom, 32511 Egypt; 7 Department of Pharmaceutical Analytical Chemistry, Faculty of Pharmacy, Menoufia University, Shebin Elkom, 32511 Egypt

**Keywords:** Chemistry, Analytical chemistry

## Abstract

Herein, a sensitive and selective spectrofluorimetric method has been developed for the determination of the ocular local anesthetic benoxinate hydrochloride (BEN-HCl) in eye drops and artificial aqueous humour. The proposed method is based on the interaction of fluorescamine with the primary amino group of BEN-HCl at room temperature. Following the excitation of the reaction product at 393 nm, the emitted relative fluorescence intensity (RFI) was measured at 483 nm. The key experimental parameters were carefully examined and optimized by adopting an analytical quality-by-design approach. The method used a two-level full factorial design (2^4^ FFD) to obtain the optimum RFI of the reaction product. The calibration curve was linear at the range of 0.10–1.0 μg/mL of BEN-HCl with sensitivity down to 0.015 μg/mL. The method was applied for analyzing the BEN-HCl eye drops and could also assess its spiked levels in artificial aqueous humour with high % recoveries (98.74–101.37%) and low SD values (≤ 1.11). To investigate the green profile of the proposed method, a greenness assessment was performed with the aid of the Analytical Eco-Scale Assessment (ESA) and GAPI. The developed method obtained a very high ESA rating score in addition to being sensitive, affordable, and environmentally sustainable. The proposed method was validated according to ICH guidelines.

## Introduction

Benoxinate hydrochloride (BEN-HCl), a *para*-aminobenzoic acid ester of 2-diethylamino ethyl-4-amino-3-butoxy benzoate^1^, is used as the hydrochloride salt in a 0.4% solution with short ophthalmological procedures^2^. The purity of BEN-HCl was 99.80 ± 0.6%^3^. The United States, European, and Japanese Pharmacopoeias all list it as an official medication when administered to the conjunctiva as a local anesthetic agent with less irritation than its analog, tetracaine^3–5^. Its analytical profile involved different techniques, including spectrophotometric^6–8^, electrochemical^9^, and chromatographic (HPLC and GC)^7,10,11^ methods. However, due to the high cost of the equipment and excessive solvent type, HPLC and GC are not often used in all laboratories; thus, other simple, rapid, and economical approaches, such as spectroscopy, are required.

In the field of material science, a candidate analytical method, namely spectrofluorimetry, has become a common base for many sensitive determinations^12–15^. Its intrinsic sensitivity, rapidity, and wide linear range of detections make the utility of spectrofluorimetry desirable for routine analysis and monitoring^16^. In this manuscript, we proposed a method for the determination of BEN-HCl based on the interaction of fluorescamine with the BEN-HCl primary amino group in a slightly alkaline pH at room temperature, which produces a strong fluorescent compound. The advantages of using fluorescamine as an amino group derivatizing fluorogenic reagent were the reason behind its use in the proposed method. Fluorescamine has several benefits over other fluorogenic compounds, including simplicity, speed, and the absence of heating requirements. Although the fluorescamine reagent is incredibly dimly fluorescent on its own, it produces a highly fluorescent reaction product (pyrrolone cation) when it reacts with an amino group^17^. This reaction is pH-dependent and extremely luminous in a slightly alkaline medium because the pyrrolone cation is unsaturated, conjugated, planar, and rigid in structure. In an acidic or strongly alkaline medium, another non-planar and less conjugated derivative is produced.

One of the main goals of analytical laboratories right now is to advance the development of green analytical chemistry (GAC). The twelve basic rules of GAC are the principles on which all greenness assessment tools depend^18–20^. The main goal of GAC is to find a balance between reducing the environmental risks connected to the analytical methodologies and reestablishing the high quality of its output. However, environmental hazards, such as harmful chemicals and/or solvents, energy-wasting machinery, the introduction of large amounts of toxic waste, or anticipated risks to the environment and human health^21,22^, are needed to be evaluated thoroughly. For this evaluation, many appraisal assessment tools have been designed^23^. The Analytical Eco-Scale assessment (ESA) and Green Analytical Procedure Index (GAPI)^24,25^ were utilized to evaluate the greenness profile of the proposed method, which was proved to be excellent green.

Furthermore, the Quality-by-Design (QbD) model makes use of a statistics-based strategy that has many advantages for designing, modifying, and validating the developed method^26,27^. Compared to univariate procedures, optimization requires significantly less effort, time, and resources. Additionally, by accurately determining significant method variables and providing plots that demonstrate the method's ideal performance and reliability, the development of experimental designs enables a better improvement and understanding of the performance of the developed method^28^. The attractiveness of QbDs arises from their capacity to identify the most critical factors, categorize them, and analyze their relationships, unlike the univariate techniques behave. The choice of a two-level full factorial design (2^4^ FFD) for this investigation was performed as it is one of the most simple screening designs, enabling the screening of many variables with a limited number of experiments^29,30^.

Hence, the proposed work aimed to create a green analytical approach with the aid of QbD that could be used to quantify BEN-HCl quickly, safely, and economically in a variety of matrices, including its pure form, eye drops, and artificial aqueous humour. Good selectivity, sensitivity, and simplicity are important features of the present method. The novelty of the current study is addressed in being the first methodology to use fluorescamine as an amino group derivatizing fluorogenic reagent for BEN-HCl by adopting a QbD approach. This study represents a green, economical, and simple analytical solution for the estimation of the studied drug without the need for large volumes of organic solvents or complicated techniques as in HPLC or LC–MS.

## Experimental

### Apparatus

Fluorescence spectra were obtained by an FS5 spectrofluorimeter (Edinburgh, UK) accessorized with a 150 W xenon lamp source for excitation and a 1-cm quartz cell. The instrument is accompanied by Fluoracle® software. The speed was 1000 nm/min, and the slit widths were chosen to be 2.0 nm. Switzerland-made analytical digital balance was used. A pH meter (Model; AD1030) from Adwa was used to measure the solutions' pH. The statistical evaluation of the experimental design was performed by Minitab® 16 statistical software (State College, Pennsylvania).

### Reagents and solutions

All the reagents and chemicals were of analytical grade. The National Organization for Drug Control and Research (NODCAR), Giza, Egypt, provided benoxinate hydrochloride (BEN-HCl) with a purity of 99.80 ± 0.6%. A 0.4%, w/v (11.6 mM) sterile ophthalmic solution (BENOX®, B. no. MF07) was purchased from a local Pharmacy.

By dissolving 10.0 mg of BEN-HCl in 100.0 mL of ultra-pure distilled water, a standard solution of BEN-HCl (0.1 mg/mL) was made. The calibration graphs and quality control (QC) samples were prepared using this solution. The quality control samples were generated at three concentration levels of 0.1, 0.4, and 1.0 μg/mL, and the calibration curve was obtained using six concentration levels in the range of 0.1–1.0 μg/mL. The solution was found to be stable for at least a week when stored in a cool and dark area.

Fluorescamine dye was purchased from Sigma-Aldrich Company (Germany). It was freshly made in acetone at a concentration of 0.04%, w/v. Boric acid and sodium hydroxide were used to prepare a borate buffer (0.1 M, pH 7.5–9). To imitate the chemical composition of human aqueous humour, artificial aqueous humour was created according to the method reported by Macri et al*.*^31^.

### Generally recommended procedure

A set of calibrated 10-mL measuring flasks was filled with precise volumes of standard BEN-HCl in the concentration range of 0.10–1.0 μg/mL. 1.5 mL of borate buffer (0.1 M, pH 8.2) and 1.0 mL of fluorescamine solution (0.04%, w/v in acetone) were added and mixed thoroughly. The volume was completed to the mark with distilled water and then left to stand for five minutes. The fluorescence of the obtained reaction product was measured at a wavelength of 483 nm after excitation at 393 nm. The same methodology was used in a blank experiment but in the absence of BEN-HCl.

### Assay of BEN-HCl in the marketed eye drops

Ultrapure distilled water was used to precisely dilute a specific volume of BENOX® ophthalmic solution, which contains 20 mg of BEN-HCl. The solution was further diluted with the same solvent to obtain a concentration of 100.0 μg/mL. Different samples within the linear range were measured following the procedure under “Generally recommended procedure”. The nominal contents of the ophthalmic solutions were calculated using the corresponding regression equation.

### Assay of BEN-HCl in the artificial aqueous humour

Artificial aqueous humour aliquots were placed in a set of 10-mL volumetric flasks (1.0 mL each). The quantitative aliquots of the BEN-HCl working solution were added within the working concentration range, followed by a 2-min vortex mix. The flasks were completed to the volume with distilled water; then, the resulting solution was filtered and analyzed as mentioned in “Generally recommended procedure”.

### Method validation

The selectivity, calibration graph linearity, limit of quantification (LOQ), limit of detection (LOD), precision, accuracy, and recovery of the method were all studied. To ascertain the selectivity, five distinct standard BEN-HCl samples spiked in the artificial aqueous humour within the linear range (0.15, 0.30, 0.40, 0.60, and 0.08 µg/mL) were measured. The linearity of the calibration curves was assessed by creating and analyzing standard BEN-HCl samples of known concentrations (over the range of 0.10–1.0 μg/mL with six concentration points) with triplicate measurements for each concentration. The LOD and LOQ were calculated using the following equations:

1 $${\text{LOD}} = {3}.{3 }\sigma /{\text{S}}$$

2 $${\text{LOQ}} = {1}0\;\sigma /{\text{S}}$$

where S is the calibration curve's slope and σ is the standard deviation of the intercept.

The accuracy and precision were assessed by determining the QC samples thrice at each of the three concentration levels (0.20, 0.40, and 1.00 µg/mL) on each of the three validation days. %RSD was used to calculate the precision, and a percentage of the measured concentration over the nominal concentration was used to calculate the accuracy. The criteria used to determine whether precision was appropriate was that the %RSD did not exceed 15%, and the accuracy was within 15% of the real value^32,33^. To determine the recovery (extraction efficiency) of BEN-HCl from the pharmaceutical preparations and/or artificial aqueous humour, the fluorescence intensity (FI) of the extracted BEN-HCl was compared to that of pure standards, which represents 100% recovery^34^.

### Factorial design

It was necessary to run initial experiments to assess the feasibility of the experimental design. After examining the impact of various experimental conditions on the fluorescence intensity of BEN-HCl, the most important independent factors were found to be buffer pH, buffer volume, fluorescamine volume, and reaction time. Two trial sets were performed, one at the maximum levels and one at the lower settings, to identify the range for each factor. The chosen range for buffer pH was (7.4–8.2), and that for buffer and fluorescamine volumes were (0.5–1.5 mL) and (0.5–1.0 mL), respectively, and the selected domain for reaction time was (0–5 min). 2^4^ FFD was performed using sixteen designed experiments to investigate the optimal settings that provide the optimal response values (Table 1).

**Table 1:** 2^4^ FFD and their dependent responses for the estimation of BEN-HCl by the developed method.

Design order				Experimental factorial design				Dependent response
Std-order	Run order	CenterPt	Blocks	Buffer pH	Buffer volume (mL)	FLC volume (mL)	Reaction time (min)	RFI
2	1	1	1	8.2	0.5	0.5	0	290
5	2	1	1	7.4	0.5	1.0	0	180
14	3	1	1	8.2	0.5	1.0	5	314
1	4	1	1	7.4	0.5	0.5	0	160
3	5	1	1	7.4	1.5	0.5	0	230
12	6	1	1	8.2	1.5	0.5	5	290
7	7	1	1	7.4	1.5	1.0	0	220
4	8	1	1	8.2	1.5	0.5	0	300
6	9	1	1	8.2	0.5	1.0	0	305
13	10	1	1	7.4	0.5	1.0	5	280
9	11	1	1	7.4	0.5	0.5	5	290
15	12	1	1	7.4	1.5	1.0	5	250
11	13	1	1	7.4	1.5	0.5	5	270
8	14	1	1	8.2	1.5	1.0	0	220
10	15	1	1	8.2	0.5	0.5	5	295
16	16	1	1	8.2	1.5	1.0	5	325

**Figure 1:**
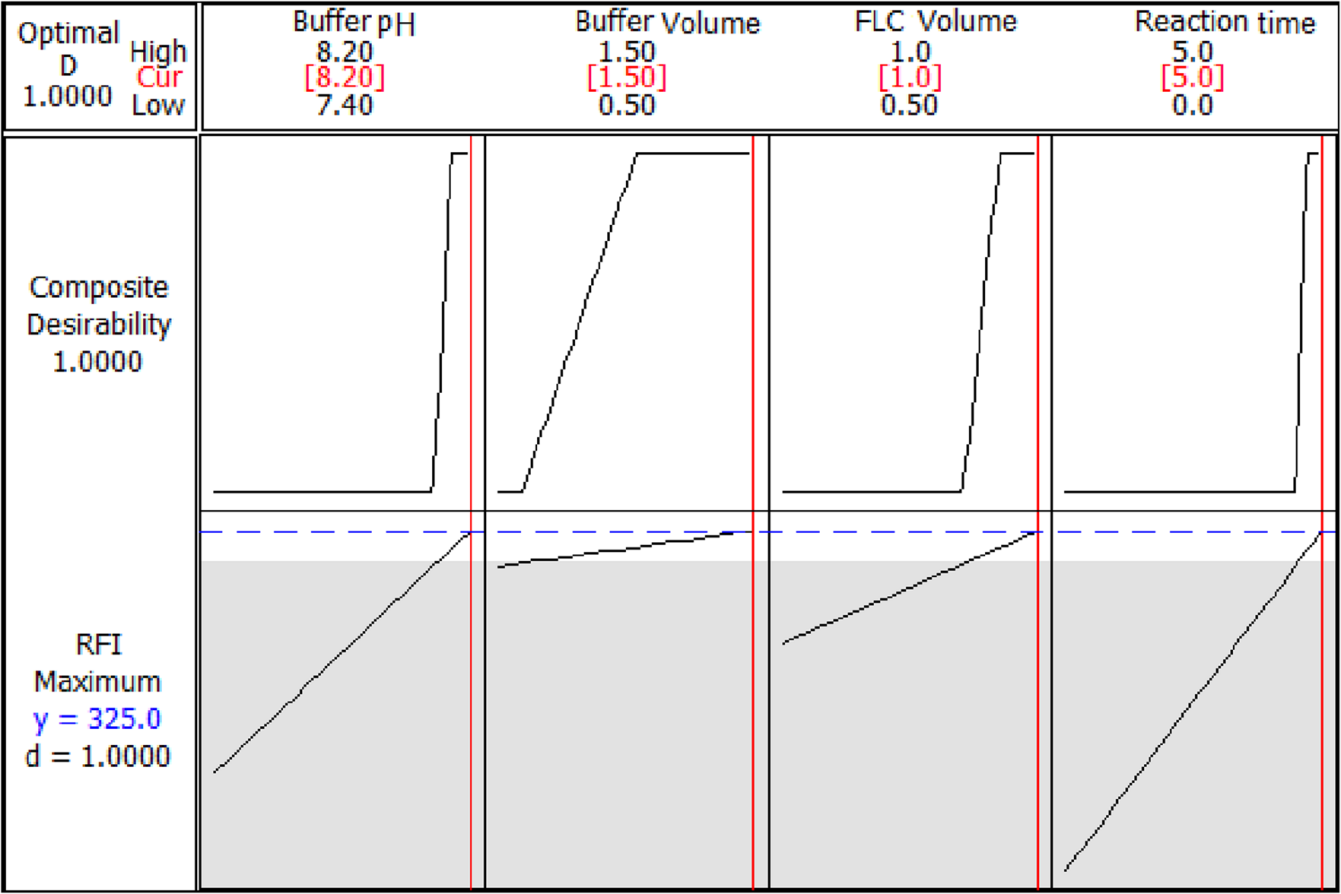
2^4^ FFD optimization plots.

The response (RFI) obtained from each experiment was measured and entered into the Minitab program. Utilizing the response optimizer, the values of both composite (D) and individual (d) desirability were maximized. Next, the most advantageous experimental parameters that produce the best response were ascertained using the optimization plot (Fig. 1). Following that, the study was conducted under the adopted ideal conditions.

## Results and Discussion

### Design and strategy for assay development

**Scheme 1:**
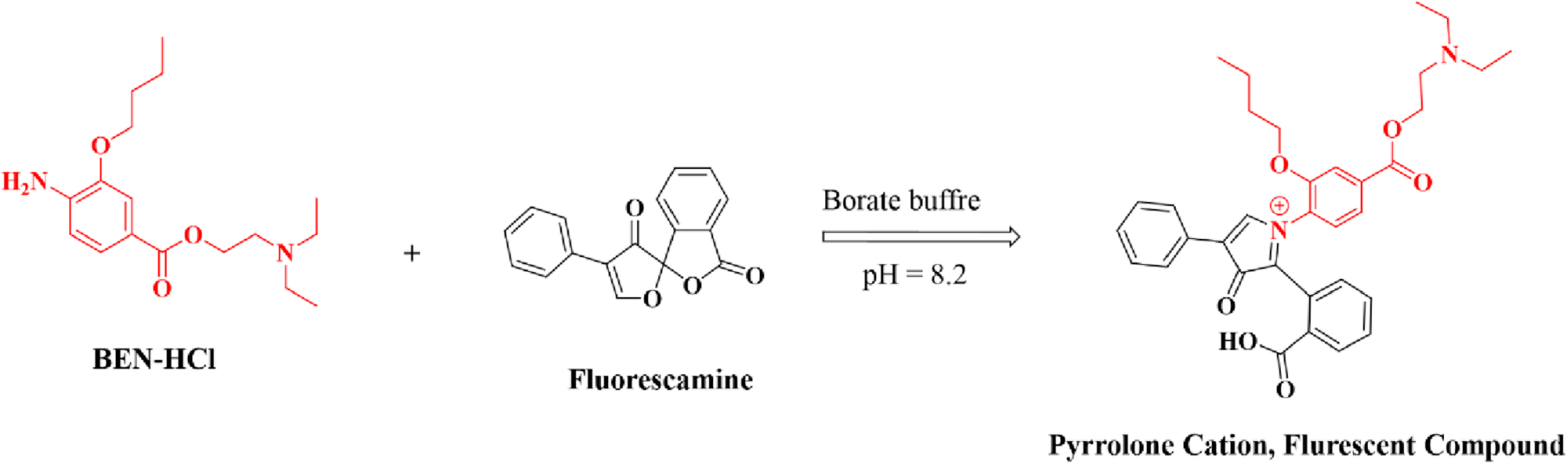
The reported reaction mechanism of BEN-HCl and fluorescamine at a pH of 8.2.

**Figure 2:**
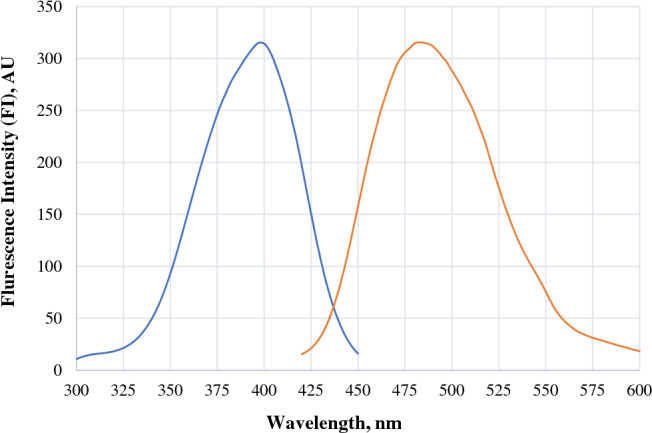
Excitation/Emission spectra of the reaction product of BEN-HCl (0.4 µg/mL), 0.1 M borate buffer (pH = 8.2, 1.5 mL), and fluorescamine (0.04%, w/v (14.0 µM), 1.0 mL).

The proposed reaction mechanism shown in Scheme 1 illustrates how BEN-HCl reacts with the reagent via its primary aliphatic amino group, stimulating the reagent's fluorescence^35–39^. The obtained fluorophore emits light at a specific wavelength of 483 nm after its excitation at 393 nm (Fig. 2).

### Optimization of the reaction conditions

According to the benefits of analytical QbD, the spectrofluorimetric properties of the fluorescent reaction product and the experimental variables that affect its stability and intensity were investigated and optimized. The buffer volume and buffer pH were shown to be the most important independent variables, while fluorescamine volume (0.04%,w/v) and reaction time were found to be less critical factors. In all the mentioned experiments, BEN-HCl was used at a concentration of 0.4 µg/mL.

**Figure 3:**
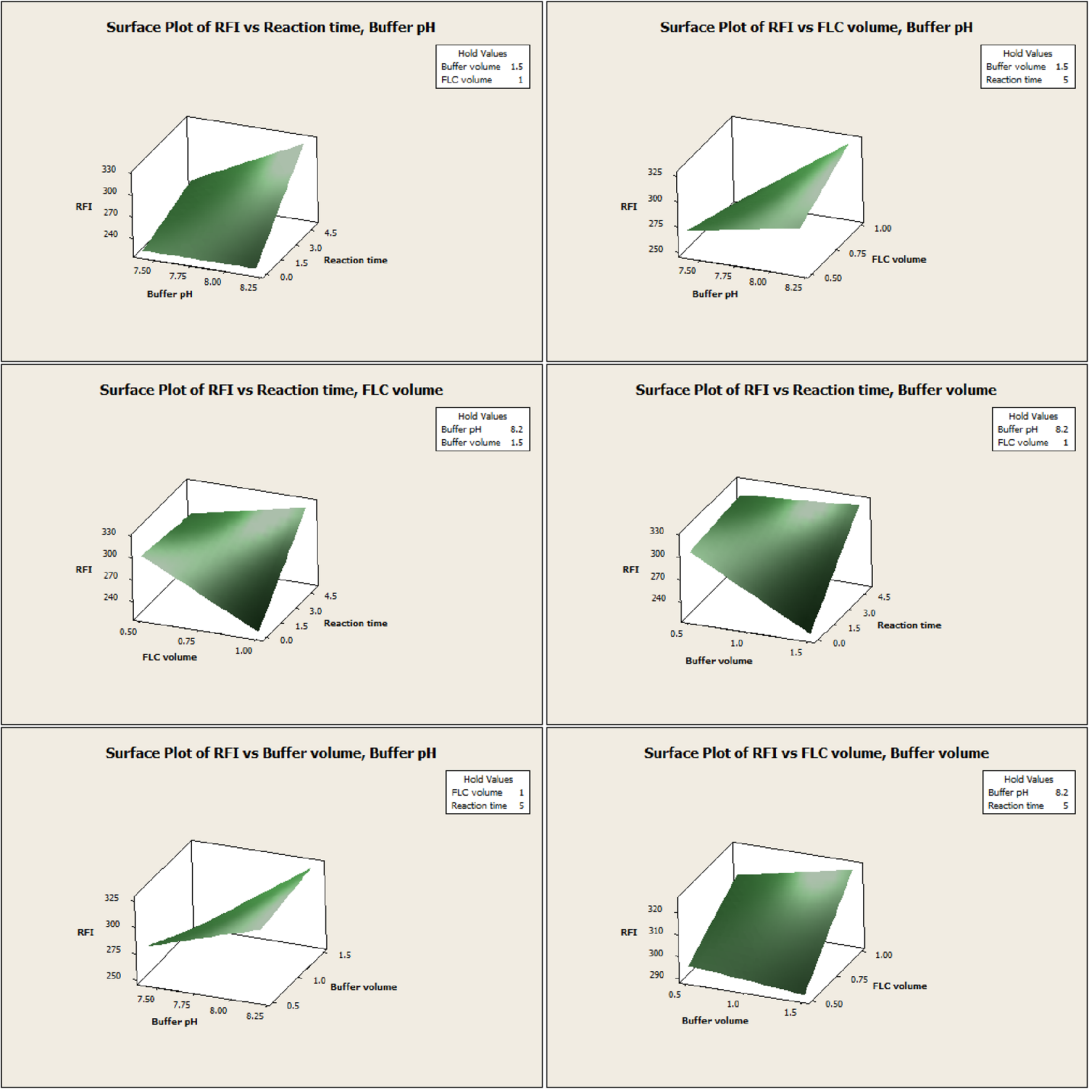
Surface plots of RFI *vs.* all pairs of significant independent factors.

From the preliminary trials, the pH effect was examined at the range of 7.0–9.2 (Fig. S1), and the intensity of the obtained product was found to develop only in a slightly alkaline medium and to vanish completely in an acidic medium due to the formation of a non-planar derivative^17^. As a result, the pH of the study was limited to the range of 7.4–8.2, which allowed the selection of the most suitable borate buffer^35–38^. The optimum pH was found to be pH 8.2 (Figs. 1 and 3). In addition, it was observed that the fluorescence intensity dropped as pH raised (Fig. S1) due to the formation of hydroxylated pyrrolone, which is non-planar and less conjugated than cationic pyrrolone with 3D structures^40^. The impact of borate buffer volume on fluorescence intensity was investigated in the initial trials in the range of 0.5–2.0 mL (Fig. S1). From the obtained results, the selected domain for the design was 0.5–1.5, and the maximum response was obtained with 1.5 mL of the buffer (Figs. 1 and 3). As a result, 1.5 mL of the prepared borate buffer with a pH value of 8.2 was employed throughout the experiment. Furthermore, a volume in the range of 0.3–1.5 of fluorescamine was tested in initial trials (Fig. S1). A volume of 0.5–1.0 mL of fluorescamine (0.04%, w/v; 14.0 µM) was selected as a domain for the design, and the maximum product fluorescence was obtained with 1.0 mL (Figs. 1 and 3). Then, the stability and formation of the final reaction product were tested periodically from 0 to 15 min in the initial trials (Fig. S1). A domain in the range of 0–5 was chosen for the design, and the optimal fluorescence intensity was achieved in approximately 5 min (Figs. 1 and 3), demonstrating how quickly the reaction product is produced, allowing increasing the method throughput analysis. The product's fluorescence was also found to be stable for at least 15 min at room temperature (Fig. S1), which adds another advantage to the developed method. These input ranges were chosen as the most significant effect on the fluorescence intensity of the studied drug was found in the selected ranges.

### Factorial design

According to the initial experiments, the four independent variables were buffer pH, buffer volume, reaction time, and fluorescamine volume, which had the greatest impact on the dependent response (RFI). After identifying the range of each variable as described under “Factorial design” in the Experimental section, 2^4^ FFD was carried out using the sixteen prepared experiments listed in Table 1. The responses from the sixteen experiments were then filled into the Minitab software, where the response optimizer was utilized to maximize the desired response (Table 2). The high composite desirability (D) score of the present study indicates that the conditions are acceptable. The optimization plot (Fig. 1) and the desirability analysis were used to recover the optimum conditions, which were found to be a pH of 8.2, a buffer volume of 1.5 mL, a fluorescamine volume of 1 mL, and a reaction time of 5 min.

**Table 2:** Response optimization of 2^4^ full factorial design for spectrofluorimetric determination of BEN-HCl.

Parameters								
	Goal	Lower	Target	Upper	Weight	Import	Predicted response	Individual desirability, (d)
RFI	Maximize	315	320	–	1	1	325	1.0
Optimum conditions: Buffer pH = 8.2, Buffer volume = 1.5 mL, FLC volume = 1 mL, Reaction time = 5 min							Composite desirability (D) = 1.0	

**Figure 4:**
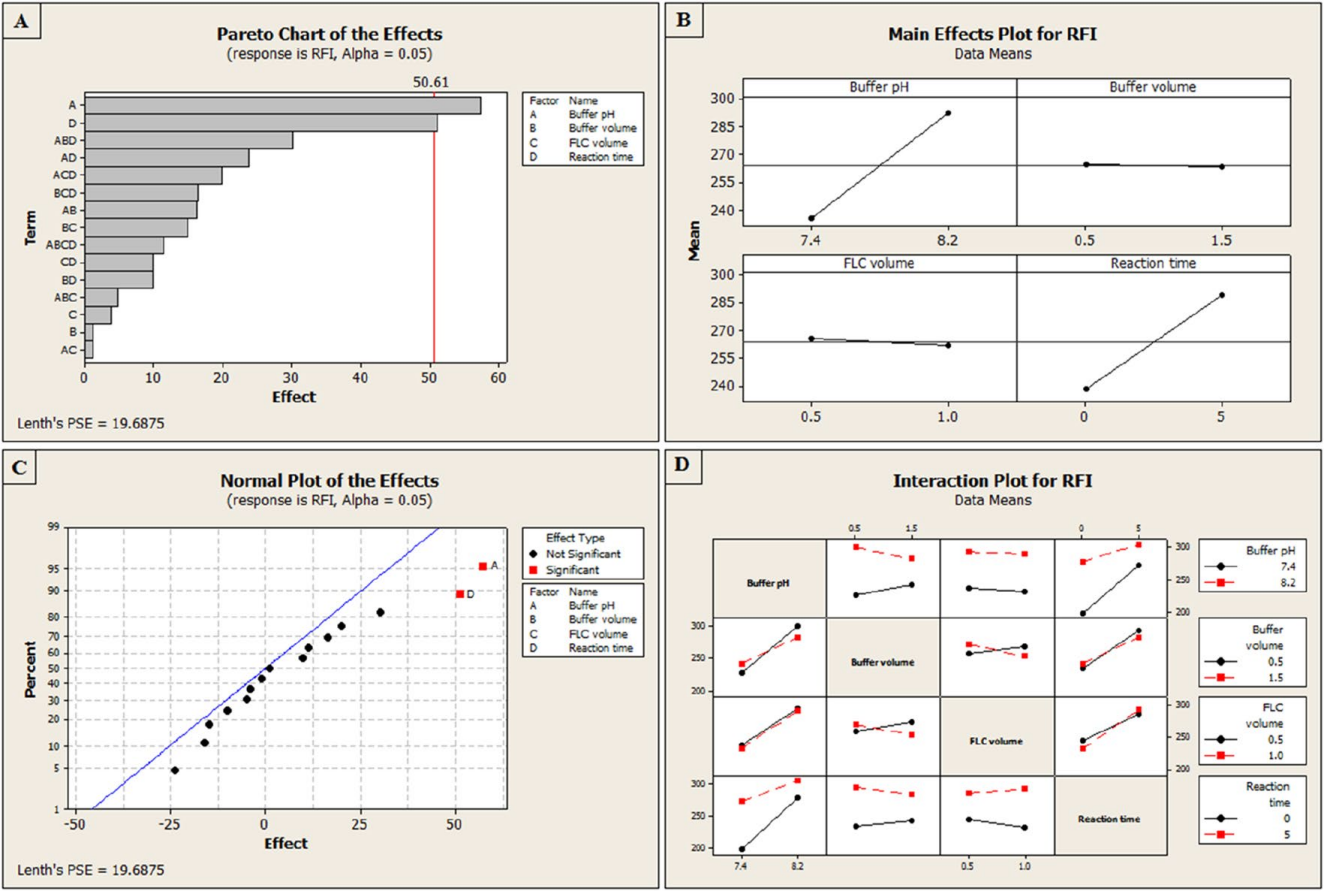
(**A**) 2^4^ FFD Pareto chart of the effects on the RFI at alpha = 0.05, (**B**) 2^4^ FFD main effects plot for RFI by data means type, (**C**) 2^4^ FFD normal plot of the effects on the RFI at alpha = 0.05, (**D**) 2^4^ FFD full interaction plots for RFI by data means type.

One of the most significant benefits of employing QbD is the ability to recognize and evaluate the most significant influencing variables on the dependent response. Further, it enables the analysis of these variables' interactions, which is not achievable using conventional optimization techniques^30^. Several Minitab plots, including the Pareto Chart, the main effects plot, the normal plot, and the full interaction plot, can be used to do this (Fig. 4). Additionally, utilizing the calculated independent variable coefficients (data in coded units) presented in Table 3 enabled the analysis of the RFI response quantitatively. It was concluded from the main effects plot, Pareto chart, and normal plot (Fig. 4) that reaction time and buffer pH have the greatest significant impact on the RFI. These characteristics have a favorable impact on the RFI, according to the estimated effect values. The interaction between buffer pH, buffer volume, and reaction time also has the most positive impact on the RFI, according to the interaction plot. In contrast, buffer volume showed the least impact on RFI and the least value among the estimated effects (Table 3). The significance of effects was also studied by the analysis of variance (ANOVA), which compares the variability of the effects with an estimation of the experimental error. The obtained results are summarized in Table 4.

**Table 3:** Estimated effects and coefficients for RFI (data in coded units).

Term	Effects^1^	Coefficients^2^	*P*-value^3^
Constant		263.69	
Buffer pH	57.37	28.69	***0.002***
Buffer volume	− 1.13	− 0.56	0.967
FLC volume	− 3.87	− 1.94	0.888
Reaction time	51.12	25.56	***0.004***
Buffer pH*Buffer volume	− 16.13	− 8.06	0.459
Buffer pH*FLC volume	1.12	0.56	0.959
Buffer pH*Reaction time	− 23.88	− 11.94	0.118
Buffer volume*FLC volume	− 14.88	− 7.44	0.590
Buffer volume*Reaction time	− 9.87	− 4.94	0.671
FLC volume*Reaction time	9.87	4.94	0.671
Buffer pH*Buffer volume*FLC volume	− 4.88	− 2.44	0.459
Buffer pH*Buffer volume* Reaction time	30.12	15.06	0.211
Buffer pH*FLC volume* Reaction time	19.87	9.94	0.123
Buffer volume*FLC volume*Reaction time	16.38	8.19	0.463
Buffer pH*Buffer volume*FLC volume*Reaction time	11.38	5.69	0.251

^1^Effects: mean the differences in the means between the high and the low levels of a factor.

^2^Coefficients: mean the differences between the marginal mean and the overall mean.

^3^Significant factors (*P*-value ≤ 0.05) appear in bold italic.

**Table 4:** Analysis of Variance (ANOVA) at 95% confidence level for RFI (data in coded units).

Source	DF	Adj SS	Adj MS
Main effects	4	23,687.7	5921.9
Buffer pH	1	13,167.6	13,167.6
Buffer volume	1	5.1	5.1
FLC volume	1	60.1	60.1
Reaction time	1	10,455.1	10,455.1
2-Way interactions	6	4990.4	831.7
Buffer pH*Buffer volume	1	1040.1	1040.1
Buffer pH*FLC volume	1	5.1	5.1
Buffer pH*Reaction time	1	2280.1	2280.1
Buffer volume*FLC volume	1	885.1	885.1
Buffer volume*Reaction time	1	390.1	390.1
FLC volume*Reaction time	1	390.1	390.1
3-Way interactions	4	6377.8	1594.4
Buffer pH*Buffer volume*FLC volume	1	95.1	95.1
Buffer pH*Buffer volume* Reaction time	1	3630.1	3630.1
Buffer pH*FLC volume* Reaction time	1	1580.1	1580.1
Buffer volume*FLC volume*Reaction time	1	1072.6	1072.6
4-Way interactions	1	517.6	517.6
Buffer pH*Buffer volume*FLC volume*Reaction time	1	517.6	517.6
Residual error	0		
Total	15	35,573.4	

*DF is degrees of freedom, SS is the sum of squares, and MS is the mean of squares.

### The stoichiometric reaction ratio

**Figure 5:**
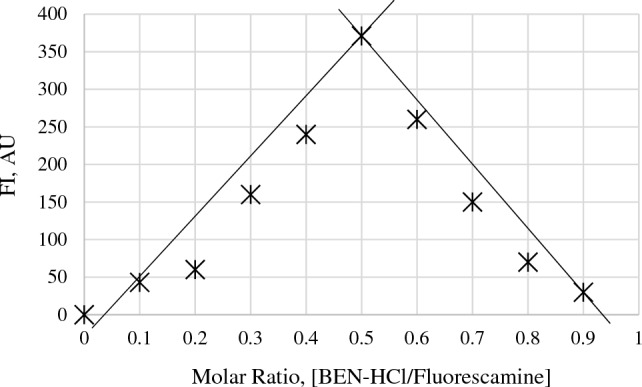
Job’s method of continuous variation of the reaction between BEN-HCl and fluorescamine (both 1.60 µM) using borate buffer, 0.1 M (1.5 mL, pH 8.2).

Applying Job's continuous variation method^41,42^ by molar ratios concentrations (1.60 µM), the ratio between the examined BEN-HCl and fluorescamine reagent was calculated. As observed, it was noted that the reaction between them had a molar ratio of 1:1 (Fig. 5). This ratio is consistent with the fact that BEN-HCl possesses one amino group.

### Method validation

The validity of the suggested method was investigated according to the International Council on Harmonization (ICH) Q2/R1 guidelines^43^; where the linearity range, LOD, LOQ, accuracy, precision, robustness, and selectivity have been established. After measuring the fluorescence intensity using the appropriate BEN-HCl concentration, the developed method exhibited acceptable linearity (r^2^ = 0.9998) in the concentration range of (0.10–1.0 μg/mL) under ideal reaction conditions. The linearity followed the regression equation y = 123.55x + 288.22. The LOD and LOQ were found to be 0.015 and 0.045 μg/mL, respectively, calculated as discussed in the experimental section.

To evaluate the method's accuracy, three levels of QC concentrations of BEN-HCl (0.20, 0.40, and 1.00 µg/mL) were used. At each concentration, triplicate measurements were performed. According to Table 5, the found % recovery range was 97.0 to 100.6, and the SD ranged from 0.58 to 1.52, indicating the high accuracy of the method.

**Table 5:** The outcomes of the proposed method's accuracy study.

Taken Conc (µg/mL)	Found Conc (µg/mL)	% Recovery ± SD (n = 3)
0.2	0.211	100.6 ± 0.58
0.4	0.389	97.0 ± 1.52
1.0	0.991	99.0 ± 1.20

For the proposed method, two levels of precision, namely, inter- and intra-day precisions, were checked. Three measurements with BEN-HCl concentrations of 0.35, 0.45, and 0.55 µg/mL were measured on the same day, and the other three tests were conducted on the following 2 days. According to Table 6, the resulting % RSD values were found to be less than 2%, demonstrating the high precision of the suggested approach.

**Table 6:** The outcomes of the proposed method's precision study.

Precision level	The taken conc., µg/mL	The found conc., µg/mL	% Recovery (n = 3)	Conc. %RSD*
Intra-day	0.35	0.359	102.01	1.52
	0.45	0.450	100.20	1.10
	0.55	0.544	99.06	0.50
Inter-day	0.35	0.357	102.78	1.15
	0.45	0.454	100.92	0.57
	0.55	0.545	99.25	0.58

*RSD relative standard deviation.

The robustness of this method was assessed by investigating the effect of minor variations of the experimental parameters on RFI, including pH (8.2 ± 0.2), buffer volume (1.5 ± 0.5 mL), and fluorescamine volume (1.0 ± 0.3 mL). The obtained values of the calculated concentration were practically expressed as % relative error (%RE). The %RE compares an experimental value to the correct or expected one, expressing the answer as the absolute value of a percentage. A %RE of 0% means that the experimental value was the same as the expected value, and the low value of it indicated the accuracy of the method as well. The intended slight variations had no effect on the RFI, demonstrating the robustness of the suggested method, as shown in Table 7.

**Table 7:** The outcomes of the proposed method's robustness study.

Variables	% Relative error (n = 3)
Buffer volume (1.5 ± 0.5 mL)	
1.0	1.0
1.5	0
2.0	0.5
pH of buffer (8.2 ± 0.2)	
8.0	1.0
8.2	0.98
8.4	0.83
Fluoroscamine volume (1.0 ± 0.3 mL)	
0.7	0
1.0	0
1.3	2.86

Next, the matrix effect was studied, where different spiked aqueous humour samples were prepared to test for the presence of any interference. The proposed method showed high % recoveries (98.74–101.37%) and low SD values (≤ 1.11) without any interference from the excipients or additives, demonstrating the negligible matrix effect (Table 8).

**Table 8:** The application of the proposed method for the determination of BEN-HCl in spiked artificial aqueous humour samples.

Parameter	The taken conc., µg/mL	The found conc., µg/mL	% Recovery ± SD (n = 3)
Spiked artificial aqueous humour	0.15	0.152	101.37
	0.30	0.302	100.62
	0.40	0.395	98.74
	0.60	0.597	99.56
	0.80	0.792	98.96
Mean	99.85		
SD (n = 3)	1.11		

### Applications

#### Application to determine the BEN-HCl in eye drops

The proposed method was successfully applied to determine BEN-HCl in its eye drops (Benox® eye drops). The average % recoveries for the various concentrations were sufficient, and there was no sign of sample matrix interference, as shown in Table 9. A statistical evaluation of the results of the suggested and reported method^7^ was performed. When using the Student's t- and F-test with a 95% level of confidence, the estimated values of both variables could not be greater than the theoretical values, as shown in Table 9.

**Table 9:** The outcomes of applying the proposed method for the determination of BEN-HCl in eye drops.

Parameter	Benox® eye drops	
	Proposed method	Comparison method^7^
% Recovery	100.20	100.10
SD (n = 3)	1.10	0.53
t-test^a^	0.14	
F-value^a^	4.30	

^a^The tabulated values at 95% confidence limit (*P* = 0.05): t = 2.306 and F = 6.338.

#### Application to BEN-HCl in artificial aqueous humour

The fixed-time method design was applied to the proposed method to examine BEN-HCl with an artificial, spiked aqueous humour. Certain BEN-HCl concentrations (0.15, 0.3, 0.4, 0.6, 0.8 μg/mL) in the range of the established calibration curve (0.1–1.0 μg/mL) were added to the prepared artificial aqueous humour, and after applying the proposed method, the RFI of each concentration was measured^31^. High % recoveries in the range of (98.74–101.37%) and low SD values (≤ 1.11), with a correlation coefficient of 0.9998, were obtained, as displayed in Table 8.

### Greenness evaluation

Analytical Eco-Scale Assessment (ESA) and Green Analytical Procedure Index (GAPI) are the relatively most popular measures because they are applicable to the majority of analytical techniques. In the current investigation, these assessment tools were used to assess the greenness profile of the developed method.

#### Analytical eco-scale assessment (ESA)

Analytical ESA, which was created primarily for the quantification of a method's green parameters, is the most useful assessment tool^44^. It depends on the calculations used to measure the penalty points that were assigned for the developed method based on the types of chemicals and solvents used, potential workplace dangers, the amount of energy used during the process, and the amount of the produced waste. A number (as the outcome of ESA) is produced by subtracting the total penalty points assigned for the method from a rating score of 100.

The tested analytical method gets greener as it gets closer to 100. The outcomes of the proposed method showed a great score of 89 when applied to the eye drops containing BEN-HCl. Consequently, the developed method has proven to be simpler and more eco-friendly. Table 10 provides a detailed description of each Analytical ESA score produced by the proposed method.

**Table 10:** Greenness assessment of the developed method using ESA and GAPI tools.

ESA			GAPI “Illustrative Pictogram”
Reagents	Items of analysis	Penalty Points	
	Fluorescamine	1	
	Amount of reagent	1	
	Acetone	4	
	Methanol	1	
Σ		***7***	
Spectrofluorimetry		1	
Waste		3	
Occupational hazards		0	
Heating temperature		0	
pH 8.2		0	
Σ		***4***	
Total penalty points (TPPs)		**11**	
ESA score	**100-TPPs**	**89**	

#### Green analytical procedure index (GAPI)

The foundation of GAPI is a three-colored phase pictogram made up of five pentagrams. The pentagram used to express each step of the analytical process symbolizes the environmental impact of that step. Three colors, green, yellow, and red, denote the degree of environmental impact. GAPI demonstrates the advantage of combining ESA's benefits because it offers both a brief overview and a thorough analysis of how environmentally friendly various steps of the analytical process are^23^. GAPI was also used to determine the green property in each step as a semi-quantitative tool. The suggested method produces little waste and needs a small amount of non-toxic chemicals. Additionally, the method is direct and is intended for qualification and quantification. The pictogram in Table 10 shows how the results, which are satisfactory, point to excellent green methodology.

## Conclusion

An efficient, rapid, sensitive, and environmentally friendly spectrofluorimetric method was developed to determine BEN-HCl in both commercial eye drops and artificial aqueous humour. The proposed technique is based on fluorescamine's interaction with the primary amino group of BEN-HCl at room temperature. At 483 nm, the RFI of the reaction product was measured after excitation at 393 nm. Adopting an analytical quality-by-design methodology allowed for careful examination and optimization of the crucial experimental parameters. The greenness profile of the developed method was verified using Analytical ESA and GAPI tools. The proposed method eliminates the shortcomings of the previously reported approaches and could be applied for the estimation of the cited drug in quality control laboratories.

## Supplementary Information

Supplementary Figure S1.

## Data Availability

The datasets generated and/or analyzed during the current study are available from the corresponding author upon reasonable request.
