# Supplementary material for: Application of quality-by-design for adopting an environmentally green fluorogenic determination of benoxinate hydrochloride in eye drops and artificial aqueous humour
Source: Sci Rep. 2023 May 26;13:8559. doi: 10.1038/s41598-023-35347-6 (PMC10219972; doi:10.1038/s41598-023-35347-6)
Supplement: Supplementary file 1 — Supplementary Figure S1. [file 41598_2023_35347_MOESM1_ESM.pdf]

Supplementary Material

Application of quality-by-design for adopting an environmentally green fluorogenic determination of benoxinate hydrochloride in eye drops and artificial aqueous humour

Mohamed A. El Hamd[1,2*], Mahmoud El-Maghrabey[3*], Galal Magdy[4*], Wael A. Mahdi[5], Sultan Alshehri[5], Amr K. A. Bass[6], Hany A. Batakoushy[7*]

[1] Department of Pharmaceutical Sciences, College of Pharmacy, Shaqra University, Shaqra 11961, Saudi Arabia

[2] Department of Pharmaceutical Analytical Chemistry, Faculty of Pharmacy, South Valley University, Qena 83523, Egypt

[3] Department of Pharmaceutical Analytical Chemistry, Faculty of Pharmacy, Mansoura University, Mansoura 35516, Egypt

[4] Pharmaceutical Analytical Chemistry Department, Faculty of Pharmacy, Kafrelsheikh University, Kafrelsheikh P.O. Box 33511, Egypt

[5] Department of Pharmaceutics, College of Pharmacy, King Saud University, Riyadh 11451, Saudi Arabia

[6] Department of Pharmaceutical Chemistry, Faculty of Pharmacy, Menoufia University, Shebin Elkom, 32511, Egypt

[7] Department of Pharmaceutical Analytical Chemistry, Faculty of Pharmacy, Menoufia University, Shebin Elkom, 32511, Egypt

*****Correspondence: Mohamed A. El Hamd: aboelhamdmohamed@su.edu.sa, Mahmoud El-Maghrabey: dr_m_hamed@mans.edu.eg, Galal Magdy: galal_magdy@pharm.kfs.edu.eg, Hany A. Batakoushy: hany.batakoushy@phrm.menofia.edu.eg

Supplementary Figures captions

Figure S1: (A) Effect of the borate buffer (0.1 M) pH and the reaction time (minutes) on the fluorescence intensity of the resulted reaction product between BEN-HCl (0.4 µg/mL, 1.160 µM) and fluorescamine (0.04%, 14.0 µM, 1.0 mL), (B) Effect of borate buffer (0.1 M, pH 8.2) volume (mL) and fluorescamine volume (14.0 µM, mL) on the fluorescence intensity of the reaction product with BEN-HCl (0.4 µg/mL, 1.160 µM).

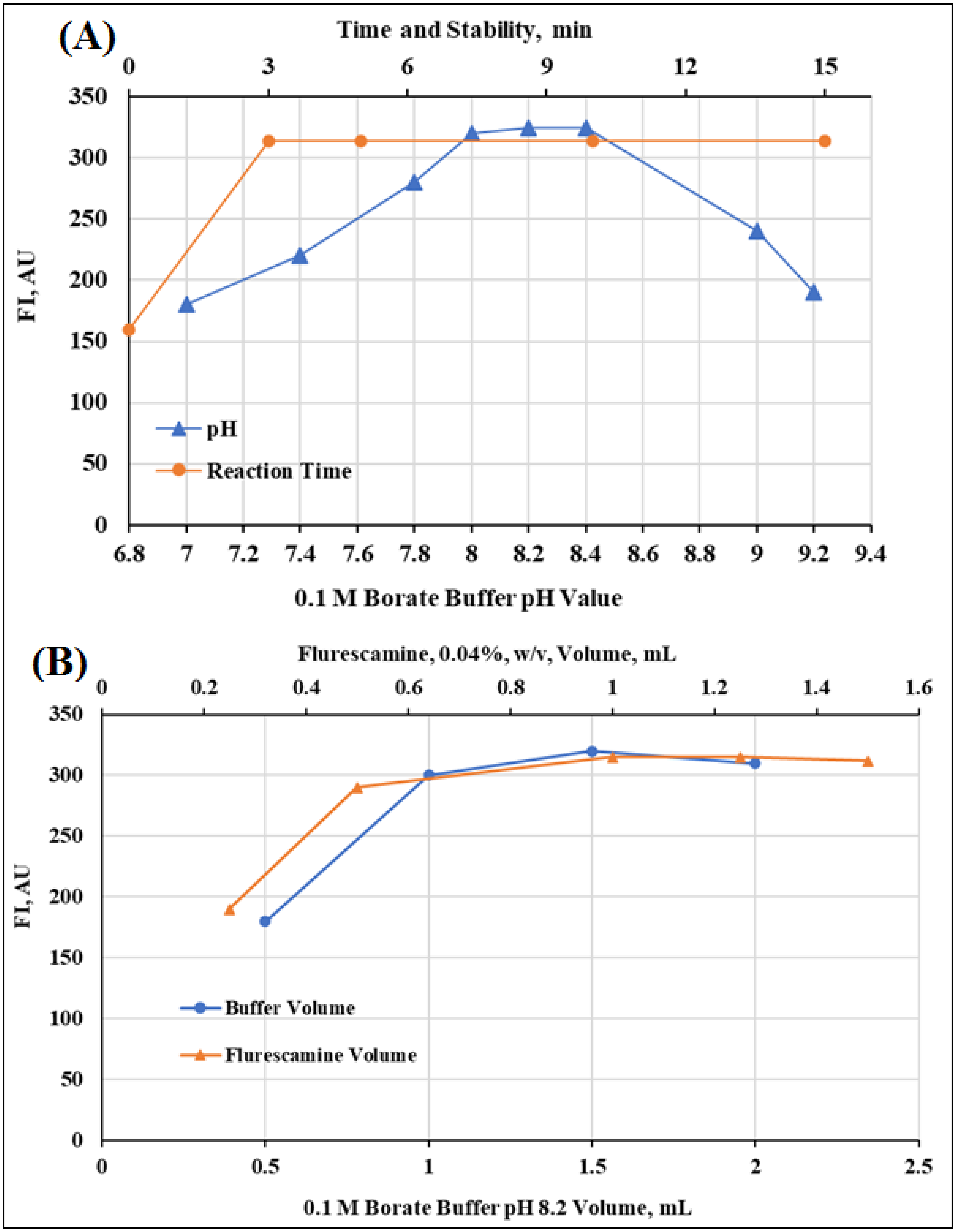
(A)
Time and Stability, min
0
3
6
9
12
15
350
300
250
200
150
100
50
0
FI, AU
pH
Reaction Time
6.8
7
7.2
7.4
7.6
7.8
8
8.2
8.4
8.6
8.8
9
9.2
9.4
0.1 M Borate Buffer pH Value
(B)
Flurescamine, 0.04%, w/v, Volume, mL
0
0.2
0.4
0.6
0.8
1
1.2
1.4
1.6
FI, AU
Buffer Volume
Flurescamine Volume
0
0.5
1
1.5
2
2.5
0.1 M Borate Buffer pH 8.2 Volume, mL

Figure S1
